# Inhibition of Astrocytic Glutamine Synthetase by Lead is Associated with a Slowed Clearance of Hydrogen Peroxide by the Glutathione System

**DOI:** 10.3389/fnint.2015.00061

**Published:** 2015-12-14

**Authors:** Stephen R. Robinson, Alan Lee, Glenda M. Bishop, Hania Czerwinska, Ralf Dringen

**Affiliations:** ^1^School of Health Sciences, RMIT UniversityMelbourne, VIC, Australia; ^2^Department of Psychology, Monash UniversityClayton, VIC, Australia; ^3^Centre for Biomolecular Interactions Bremen and Centre for Environmental Research and Sustainable Technology, Faculty 2 (Biology/Chemistry), University of BremenBremen, Germany

**Keywords:** astrocytes, glutamine synthetase, glutathione, glutamate, oxidative stress, toxicity

## Abstract

Lead intoxication in humans is characterized by cognitive impairments, particularly in the domain of memory, where evidence indicates that glutamatergic neurotransmission may be impacted. Animal and cell culture studies have shown that lead decreases the expression and activity of glutamine synthetase (GS) in astrocytes, yet the basis of this effect is uncertain. To investigate the mechanism responsible, the present study exposed primary astrocyte cultures to a range of concentrations of lead acetate (0–330 μM) for up to 24 h. GS activity was significantly reduced in cells following 24 h incubation with 100 or 330 μM lead acetate. However, no reduction in GS activity was detected when astrocytic lysates were co-incubated with lead acetate, suggesting that the mechanism is not due to a direct interaction and involves intact cells. Since GS is highly sensitive to oxidative stress, the capacity of lead to inhibit the clearance of hydrogen peroxide (H_2_O_2_) was investigated. It was found that exposure to lead significantly diminished the capacity of astrocytes to degrade H_2_O_2_, and that this was due to a reduction in the effectiveness of the glutathione system, rather than to catalase. These results suggest that the inhibition of GS activity in lead poisoning is a consequence of slowed H_2_O_2_ clearance, and supports the glutathione pathway as a primary therapeutic target.

## Introduction

A primary function of astrocytes is to recycle synaptically-released glutamate (Pow and Robinson, 1994). Unlike neurones, astrocytes express glutamine synthetase (GS; Norenberg and Martinez-Hernandez, 1979; Ong et al., 1993; Robinson, 2000), which catalyzes the ATP-dependent condensation of glutamate and ammonia to form glutamine (Rose et al., 2013), which is subsequently released for uptake by neurones and deamidation to glutamate, in the glutamate-glutamine cycle (Westergaard et al., 1995; Hertz et al., 1999). GS is particularly sensitive to inactivation by iron-mediated oxidative stress (Fernandes et al., 2011), and consequently depleted GS activity levels in tissue have often been used as an indicator of oxidative stress (Schor, 1988; Robinson, 2000).

Studies have linked the toxicity of lead to elevated oxidative stress, with lead exposure correlating with increased production of free radicals and a lowering of antioxidant reserves (Patrick, 2006; Rubino, 2015). In the brain, astrocytes contribute to the defense against toxic metals, xenobiotics and oxidative stress (Dringen et al., 2014). Hydrogen peroxide (H_2_O_2_), a common source of oxidative stress, is generated during erobic metabolism by the action of superoxide dismutases and several oxidases. H_2_O_2_ is broken down in iron-catalyzed reactions to form hydroxyl radicals, which react readily with proteins, lipids and DNA, and are thus toxic to cells. To limit free radical formation from H_2_O_2_, astrocytes utilize two main antioxidant systems. The first of these, catalase, rapidly degrades H_2_O_2_, even when the peroxide is applied acutely at high concentrations (Dringen and Hamprecht, 1997). The rate of H_2_O_2_ breakdown is significantly slowed by pre-incubating astrocytes with the catalase inhibitor 3-amino-1,2,4-triazole (3AT), and this is correlated with an increase in cell death (Liddell et al., 2006).

The second antioxidant system is the glutathione pathway, whereby H_2_O_2_ is reduced to water in a reaction catalyzed by glutathione peroxidase (GPx). Glutathione (GSH) serves as the electron donor, and is itself oxidized to glutathione disulfide (GSSG). GSSG is reduced to GSH through the action of glutathione reductase (GR; Dringen and Hamprecht, 1997; Dringen et al., 2014). Astrocytes pre-incubated with buthionine sulfoximine (BSO), a GSH synthesis inhibitor, show a marked reduction in their rate of H_2_O_2_ clearance and an increase in cell death (Liddell et al., 2006). Application of 3AT and BSO to inhibit both antioxidant systems, futher retards H_2_O_2_ clearance rates and exacerbates cell death (Liddell et al., 2006). The high cellular GSH content of astrocytes, combined with their efficiency in breaking down peroxides, protects astrocytes and neighboring cells from oxidants and toxins (Dringen et al., 2014).

While several animal and cell culture studies have shown that application of lead diminishes the expression and activity of GS (Engle and Volpe, 1990; Sierra and Tiffany-Castiglioni, 1991), the basis of this effect is uncertain. The decrease in GS activity may be due to lead binding to cysteine residues on GS, thereby interfering with its catalytic activity (Tang et al., 1996; Qian et al., 1999). Alternatively, GS may be inactivated by reactive oxygen species that result from a diminished effectiveness of the GSH system. Lead binds to sulfhydryl groups, giving it an affinity for GSH which then cannot act an antioxidant (Patrick, 2006). Lead can also bind to the catalytic site of GR, irreversibly inhibiting the enzyme and preventing it from reducing GSSG to GSH (Rubino, 2015). Impairment of the glutathione system will decrease cellular antioxidative capacity and reduce protection from oxidative stress (Scortegagna et al., 1998; Aykin-Burns et al., 2003).

It is possible that lead binds to the active site of GS inhibiting it directly, or the inhibition of lead may be a downstream event, secondary to inactivation of the glutathione system. The present study conducted experiments to discriminate between these alternatives. Primary astrocyte cultures were exposed to a range of concentrations of lead for up to 24 h. We confirmed that GS activity is significantly lowered in cultures following incubation with lead acetate. However we found that this effect is not replicated when astrocyte lysates are exposed to lead, suggesting that, when in the presence of other cellular components, lead does not directly interfere with the catalytic activity of GS. We also demonstrated that lead limits the capacity of astrocytes to degrade H_2_O_2_, and that this appears to be due to an impairment of the GSH system.

## Materials and Methods

### Materials

This study was carried out in accordance with the guidelines of the National Health and Medical Research Council (NHMRC) of Australia. The protocol was approved by Monash University’s Psychology Animal Ethics Committee. Primary astrocyte cell cultures were derived from newborn Wistar rat pups obtained from Monash Animal Services. Constituents of growth media were obtained from Gibco (Carlsbad, CA, USA): Dulbecco’s modified Eagle medium (DMEM), fetal calf serum (FCS), streptomycin sulfate and penicillin G. Triton X-100 was obtained from Ajax Finechem (Seven Hills, Australia). Lead (II) acetate trihydrate, BSO, 3AT, H_2_O_2_ and all other chemicals were obtained from Sigma (Australia). Twenty four-well cell culture dishes and 96-well microtiter plates were obtained from Greiner Bio-One (Frickenhausen, Germany).

### Cell Cultures

Primary astrocyte cultures were obtained from the brains of newborn Wistar rat pups (<24 h old) as previously described (Hamprecht and Löffler, 1985). Astrocytes were seeded at approximately 3 × 10^5^ cells/well in 24-well culture plates. Cells were grown in humidified incubators (Heraeus Instruments) at 10% CO_2_. Growth medium was replaced every 6th or 7th day until cultures were confluent, at which time they were used for experimentation (14–21 days).

### Cell Viability and Protein Content

The activity of lactate dehydrogenase (LDH) released by cells into media by treated cells was measured to determine the extent of cell death, as previously described (Dringen et al., 1998). Hundred percent cell death values were derived from cells lysed with Triton X-100. Cellular protein content per well was determined via the Lowry method (Lowry et al., 1951).

### GS Activity

GS activity was measured with a colorimetric assay (Fernandes et al., 2011). To determine the effect of lead on GS activity, cultures were incubated with 0, 33, 100 or 330 μM lead acetate in DMEM for 2 or 24 h. Cells were washed with ice-cold PBS and frozen at −20°C for 30 min before being warmed to 37°C and lysed with 200 μl of 50 mM imidazole/ HCl buffer (IHB), pH 7.2. After 5 min, 200 μl of a reaction mix was added to the lysates (50 mM L-glutamine, 2 mM manganese chloride, 25 mM sodium arsenate, 0.16 mM ADP and 25 mM NH_2_OH*HCl). Following 30 min incubation with the reaction mix, 800 μl of a solution of 0.37 M ferric chloride (FeCl_3_), 0.67 M HCl and 0.2 M trichloroacetic acid was added to halt the reaction. Samples were transferred to microfuge tubes and centrifuged for 5 min at 15000 g. Three hundred microliters of the supernatant was transferred to a microtiter plate. Absorbances were measured spectrophotometrically (Multiskan Ascent plate reader, Thermo Labsystems) at 500 nm and the samples were compared to standard solutions of the reaction product γ-glutamylhydroxymate (0 and 1000 nmol), which had been processed identically to the samples.

To examine the direct effect of lead on GS activity, a variation of the method of (Santoro et al., 2001) was performed on lysed astrocytes. Since 10 μM lead is reported to produce direct and complete inhibition of GS (Sierra and Tiffany-Castiglioni, 1991), low concentrations of lead acetate were prepared to test the concentration-dependency of lead inhibition: 0, 2.5, 5, 7.5 and 10 μM. To allow comparison with the incubations on live cultures, lead concentrations of 33, 100 and 330 μM were also analyzed. Untreated cell culture plates were washed, frozen and brought to 37°C as described above, then lysed with 100 μl of 100 mM IHB for 5 min. One Hundred microliters of lead acetate in IHB was added to achieve the final concentrations listed above and the lysate was incubated for a further 5 min. Two hundred microliters of GS reaction mix was then applied for 30 min, with the remaining steps as described for lead-incubated cell cultures.

For cell culture and lysed cell experiments, final GS activity values were standardized against protein samples from cells or lysates incubated with equivalent lead acetate concentrations, thereby correcting for the loss of GS due to cell death. GS activity was then expressed as a percentage of the control values (the 0 μM lead acetate treatments), to provide values for specific GS activity.

### H_2_O_2_ Clearance

In control cultures, the H_2_O_2_ clearance system was examined independently of lead, including conditions that partially inhibited H_2_O_2_ clearance for subsequent comparison to the effects of lead. 3AT completely inhibits catalase activity within 2 h, whereas BSO requires 24 h to deplete GSH to 14% of control levels (Dringen and Hamprecht, 1997). Therefore, the BSO-containing treatments were pre-incubated for 18 h with BSO in DMEM followed by a further 6 h with the addition of 3AT and/or lead acetate, as appropriate. For comparability, conditions not containing BSO received a 18 h pre-incubation in DMEM only. Similarly, to study the effect of lead on H_2_O_2_ clearance, lead-treated cultures were pre-incubated for 18 h with DMEM only, then incubated with 0, 10, 33, 100 or 330 μM lead acetate for 6 h, with or without the addition of 10 mM 3AT, prior to the addition of H_2_O_2_.

For each replication of this experiment, the incubations were carried out on three sets of cells. One set was used for the H_2_O_2_ clearance assay, the second set for protein estimates to standardize H_2_O_2_ clearance values, while the third set was used to provide a 100% cell death control condition for the LDH assay (Dringen et al., 1998).

The clearance of H_2_O_2_ was determined as previously described (Dringen et al., 1998). After the incubations described above, culture media was collected for the subsequent measurement of released LDH. Cells were washed with incubation buffer (IB; 20 mM HEPES, 145 mM NaCl, 0.8 mM Na_2_HPO_4_, 5.4 mM KCl, 1 mM MgCl_2_, 1.8 mM CaCl_2_ and 5 mM glucose, pH 7.4) before adding a bolus of 500 μl IB containing 100 μM H_2_O_2_. Ten microliters samples were taken from each well after 2, 4, 6, 8, 10, 20 and 60 min and transferred to microtiter wells containing 170 μl 25 mM sulfuric acid to halt further degradation of H_2_O_2_. One hundred and eighty microliters of a freshly made reaction mixture (100 mM sorbitol, 25 mM H_2_SO_4_, 0.25 mM (NH_4_)_2_Fe(SO_4_)_2_ and 100 μM xylenol orange) was added to each well for 45 min. The resulting color change was proportional to the concentration of H_2_O_2_ remaining in the sample. Absorbances were measured at 550 nm and compared to standards of known H_2_O_2_ concentration.

To compare rates of H_2_O_2_ degradation across treatments and cultures, results were standardized against protein values, and half-times were calculated as previously described (Dringen et al., 1998). Specific detoxification rate constants (*D*-values) were calculated by taking the inverse of the product of protein content and the half-time of H_2_O_2_ clearance. The *D*-values represent the concentration of H_2_O_2_ that is cleared per minute, per mg of protein.

### Statistical Analysis

Each treatment condition and time point was performed in triplicate and replicated on astrocyte cultures or lysates derived from three independently prepared cultures. Data provided in the results section represent mean ± SD. Values for GS activity in cell cultures and cell lysates were analyzed using one-way independent samples ANOVAs, with *post hoc* corrections, to determine the effect of each lead concentration on GS activity at each time point. Such analyses were also applied to data obtained from the same cultures and processed through the LDH cell death assay.

*D*-values calculated after H_2_O_2_ detoxification experiments were analyzed with one-way independent samples ANOVAs with treatment type (BSO, DMEM, BSO/3AT, 3AT, 10 μM lead/3AT, 33 μM lead/3AT, 100 μM lead/3AT, 330 μM lead/3AT, 10 μM lead, 33 μM lead, 100 μM lead or 330 μM lead) being the independent factor.

## Results

### Effect of Lead Acetate on Specific GS Activity and Cell Viability

**Figure 1:**
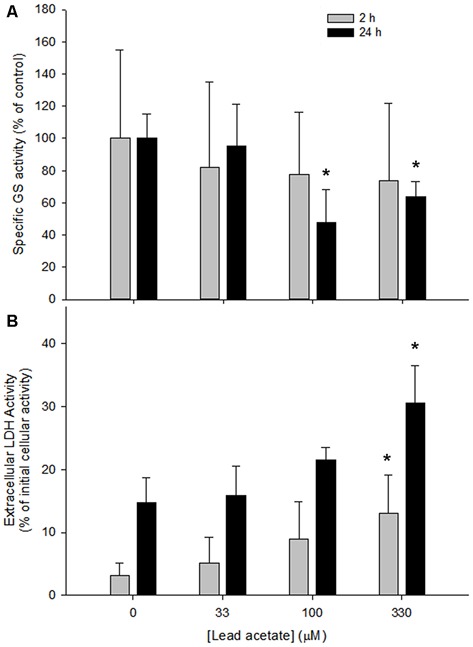
**Specific glutamine synthetase (GS) activity and cell viability in rat astrocyte cultures after 2 or 24 h incubation with four concentrations of lead acetate (0, 33, 100 and 330 μM).** **(A)** Specific GS activity expressed as a percentage of that in untreated cells. Each bar represents mean ± SD of *n* = 9 samples. **(B)** Cell death as determined by activity of Lactate dehydrogenase (LDH) released into the media. Each bar represents means ± SD of *n* = 6 samples. *Significant differences between lead-treated and untreated cells at their respective timepoint (*p* < 0.05).

In cultures treated with 0–330 μM lead acetate for 2 h, no significant differences in specific GS activity were observed between the control treatment and any of the lead concentrations (*F*_(3,32)_ = 0.396, *p* > 0.05). However, after 24 h, cells treated with 100 or 330 μM lead acetate displayed a marked reduction in specific GS activity (40–50%) when compared to control cells (*F*_(3,32)_ = 11.052, *p* > 0.05; Figure 1A).

Cell viability was examined after incubation with lead. After 2 h, 330 μM lead acetate caused a modest yet significant increase in LDH release when compared to untreated cells and the other lead acetate concentrations (*F*_(16,19)_ = 6.415, *p* < 0.05; Figure 1B). By 24 h, 330 μM lead had caused a doubling of LDH release (*F*_(18,21)_ = 15.762, *p* < 0.05; Figure 1B), whereas values for 33 and 100 μM lead exposure did not differ significantly from controls. The detectable activity of extracellular LDH showed a remarkable linear correspondence as a function of lead concentration at both time points examined. Thus at 2 h the correlation coefficient was *r* = 0.957 and at 24 h the correlation coefficient was *r* = 0.990.

**Figure 2:**
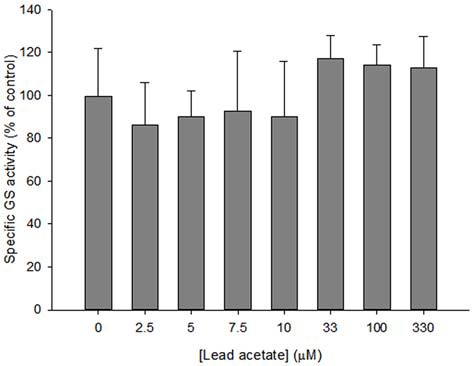
**Specific GS activity in astrocyte lysates incubated with 0–330 μM lead acetate for 35 min.** Bars show means ± SD of *n* = 6 samples. No significant difference was found between control and treated lysates.

Specific GS activity in astrocyte lysates was examined after treatment with lead acetate. Compared to controls (0 μM lead), no significant reduction of GS activity was found in lysates for any lead acetate concentration (*F*_(9,61)_ = 1.714, *p* > 0.05; Figure 2).

### Effect of Lead Acetate on H_2_O_2_ Clearance by Astrocytes

**Figure 3:**
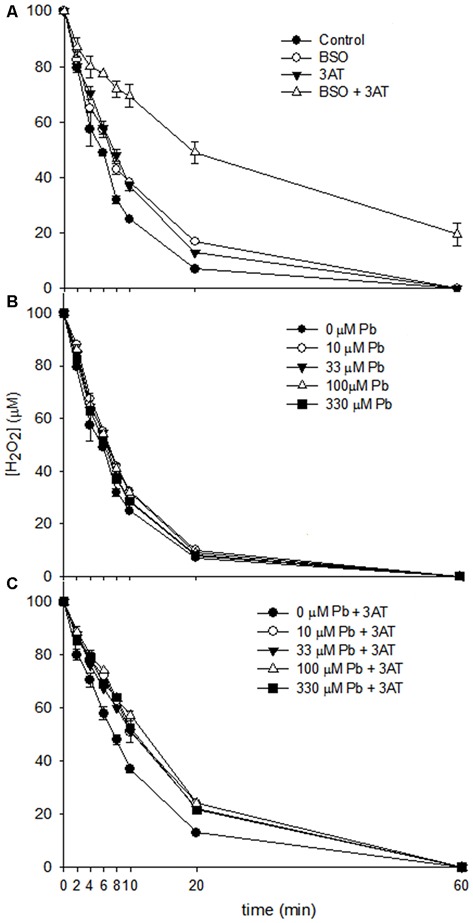
**Clearance of H_2_O_2_ by rat astrocyte cultures.** Cells were incubated for 60 min with 500 μl of 100 μM H_2_O_2_, and media were collected at the specified time points for measurement of H_2_O_2_ concentration. **(A)** Dulbecco’s modified eagle medium (DMEM) only control, 1 mM Buthionine sulfoximine (BSO), 10 mM 3AT or 1 mM BSO + 10 mM 3AT. **(B)** 10–330 μM lead acetate. **(C)** 10–330 μM lead acetate + 10 mM 3AT. Each data point represents mean ± SD of *n* = 9 samples.

The influence of lead on the capacity of astrocytes to degrade H_2_O_2_ was examined. The peroxide clearance curves (Figure 3) revealed that in all conditions investigated, except for BSO + 3AT (Figure 3A), all of the H_2_O_2_ applied was cleared within 60 min. However, the rates of peroxide clearance differed between conditions. While cultures treated with lead acetate demonstrated a slightly slower rate of peroxide clearance in the first 20 min compared with control cells (Figure 3B), the rates of clearance were slowed substantially when the cells had been exposed to both lead and the catalase inhibitor 3AT (Figure 3C), indicating an additive effect.

**Figure 4:**
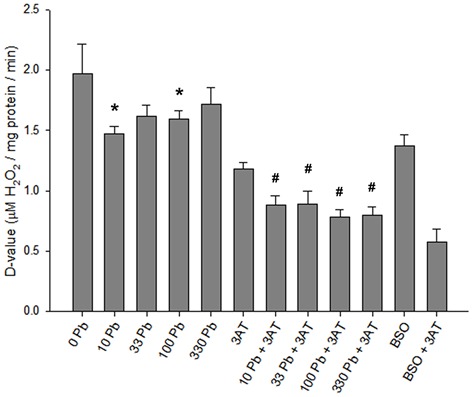
***D*-values for H_2_O_2_ clearance in rat astrocyte cultures.** Standardized H_2_O_2_ clearance values were derived from H_2_O_2_ half-times. The higher the *D*-values, the faster the rate of detoxification. Incubation conditions were the same as in Figure 3. Treatment concentrations: 1 mM BSO, 10 mM 3AT; concentrations for lead (Pb) are in μM. Statistical analyses were performed on lead-treated conditions. Significant differences (*p* < 0.05) between lead-treated and untreated control are represented by *. Significant differences (*p* < 0.05) between lead + 3AT-treated and 3AT-treated controls are represented by ^#^. Each bar represents a mean ± SD of *n* = 9 samples, except for those treated with 10 μM lead where *n* = 6.

Analysis of specific detoxification rate constants (*D*-values; Figure 4), derived from the half-times of extracellular H_2_O_2_ degradation and the specific protein values, revealed a significant effect of the treatments (*F*_(11,87)_ = 148.180, *p* < 0.05). Exposure of astrocytes to 10 or 100 μM lead acetate for 6 h significantly slowed the rates of H_2_O_2_ clearance when compared to astrocytes treated without lead (Figure 4). Furthermore, all of the lead + 3AT treatments yielded significantly slower rates of H_2_O_2_ detoxification than treatment with 3AT alone, but faster rates than those observed after treatment with BSO + 3AT (Figure 4). The *D*-values of astrocytes treated with different lead concentrations did not differ significantly from each other (Figure 4, columns 2–5). Therefore, increasing lead concentrations do not cause greater impairment of peroxide clearance. Under the conditions investigated, the lowest lead concentration applied (10 μM) caused maximal effects on peroxide clearance in both the presence and the absence of 3AT.

### Toxicity of Treatments with H_2_O_2_

**Figure 5:**
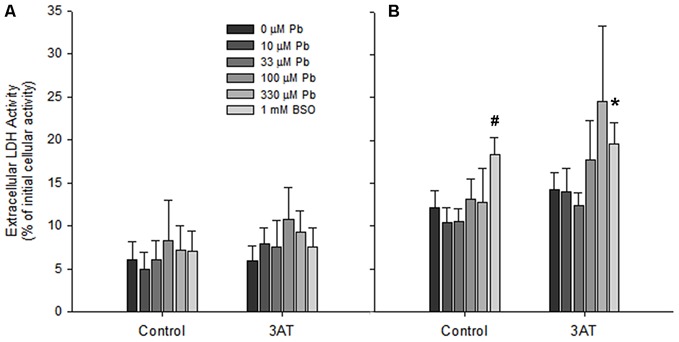
**Cell viability in rat astrocyte cultures before and after 100 μM H_2_O_2_ treatment.** Incubation conditions were the same as in Figure 3. **(A)** Extracellular LDH immediately before application of 100 μM H_2_O_2_. **(B)** Extracellular LDH 60 min after application of 100 μM H_2_O_2_. Significant differences (*p* < 0.05) between treatments and the untreated controls are represented by ^#^ for the no-3AT control group, and by * for the 3AT group. Each bar represents a mean ± SD of *n* = 9 samples, except for those treated with 10 μM lead where *n* = 6.

Prior to treatment with a bolus of H_2_O_2_, the amount of LDH released by cultures exposed to lead and/or to 3AT was within the normative range for all treatments (less than 10% LDH release), indicating that the treatments did not cause significant cell death (Figure 5A). However, after 60 min incubation with 100 μM H_2_O_2_, LDH levels were elevated in all conditions (Figure 5B). When these levels were analyzed, a one-way ANOVA indicated a significant effect of treatment (*F*_(11,90)_ = 12.052, *p* < 0.05). Dunnett’s T3 *post hoc* analyses demonstrated that none of the lead treatments significantly increased the extent of cell death compared to the respective control condition (*p* > 0.05). However, after 60 min incubation with H_2_O_2_, the BSO-treated group demonstrated a significant increase in LDH release, both in the presence of 3AT (*T3* = 7.483 + 1.073, *p* < 0.05) and in the absence of 3AT (*T3* = 6.207 + 0.930, *p* < 0.05; Figure 5B).

## Discussion

While lead intoxication is known to impair GS activity, uncertainty exists regarding the basis of this impairment. It is possible that lead binds to the active site of GS inhibiting it directly, or the inhibition of lead may be a downstream event, secondary to inactivation of the glutathione system. The present study conducted experiments to discriminate between these alternatives. It has been shown that low concentrations of lead are capable of reducing GS activity in astrocytes that have been cultured with lead for a week or more (Engle and Volpe, 1990; Sierra and Tiffany-Castiglioni, 1991). The present study investigated whether astrocytes incubated with lead for shorter periods (2 or 24 h) also show an altered GS activity. Our results revealed that the effect of lead on specific GS activity in cultured astrocytes is dose- and time-dependent, with 100 and 330 μM lead acetate significantly reducing GS activity following 24 h incubation, but not after 2 h. The slowness of this effect is inconsistent with a direct inactivation of GS by lead. For instance, direct inhibitors of GS such as methionine sulfoximine can inhibit GS activity in intact tissue within minutes (Barnett et al., 2000).

Previous studies have speculated that lead might bind to cysteine residues on GS, thereby directly impairing the function of this enzyme (Sierra and Tiffany-Castiglioni, 1991; Tang et al., 1996). This possibility was investigated in the present study by lysing astrocyte cultures to release their GS and subsequently incubating the lysates with lead for 35 min. It was expected that the inhibitory effect of lead would be rapid and would require lower concentrations than those effective on living astrocytes, which require time to accumulate lead. The present results, however, demonstrated that lead does not inhibit GS activity in cell lysates. Regardless of the concentration of lead used, GS activity did not differ from control lysates. These results are inconsistent with an earlier report (Sierra and Tiffany-Castiglioni, 1991) that direct application of 10 μM lead is sufficient to maximally inhibit the activity of GS in astrocyte lysates within 30 min. Those researchers used a radio ligand assay for GS activity, whereas we used a colorimetric assay, testing for an enzymatic side reaction of GS which appears to be unaffected by lead. Regardless of the reason for this discrepancy, the present results imply that the inhibition of GS is not caused by lead binding to cysteine residues at the active site of the enzyme, and instead may be an indirect consequence of a secondary mechanism.

While studies have noted that lead toxicity is accompanied by oxidative stress, there is uncertainty regarding how lead promotes this, since lead itself is not redox-active. Much of the evidence points to the fact that lead has an affinity for sulfhydryl groups and hence may bind readily to GSH and enzymes in the GSH system (Patrick, 2006; Rubino, 2015), thereby reducing the cellular capacity to clear H_2_O_2_ (Tang et al., 1996). For example, the blood and tissues (including brain) of mice exposed to toxic concentrations of lead demonstrate significantly lower levels of endogenous GSH (Penugonda and Ercal, 2011). The results of the present study indicate that when compared to lead-free controls, lead acetate decreased H_2_O_2_ clearance rates, and that the extent of slowing was about half that caused by the GSH synthesis inhibitor BSO, regardless of the presence or absence of 3AT in the system. These observations are consistent with the hypothesis that lead targets the GSH system by affecting the synthesis and regeneration of GSH. By slowing the rate of detoxification, metabolically-produced H_2_O_2_ will remain in cells for a longer period, giving it an increased potential to cause oxidative stress and to oxidize GS. It should be noted however, that GS activity in astrocytes is unaffected by the presence of H_2_O_2_ alone; inactivation of GS requires the presence of iron (Fernandes et al., 2011).

Compounds that block GS activity cause profound memory impairments and cognitive deficits (e.g., Gibbs et al., 1996; reviewed by Robinson, 2000). The glutamate-glutamine cycle can be interrupted by inhibiting astrocytic GS with methionine sulfoximine (Pow and Robinson, 1994). The subsequent depletion of glutamate stores and interruption of glutamatergic neurotransmission occurs with surprising rapidity. For instance, 40 mM doses of methionine sulfoximine delivered to the retina of rats results in blindness within 2 min, which can be reversed by the administration of glutamine (Barnett et al., 2000). Similarly, delivery of methionine sulfoximine into the hyperstriatum of chicks prevents memory consolidation, an effect that is reversed by the administration of glutamine, demonstrating the essential contribution of astrocytic GS to learning and the consolidation of memories (Gibbs et al., 1996; Hertz et al., 1996; Gibbs and Hertz, 2005). In rats the inhibition of GS impairs the temporal component of memories (Kant et al., 2014).

Verbal and nonverbal memory is impaired in individuals who have been chronically exposed to lead, with the greatest deficits being evident in children, particularly with respect to their global intelligence and capacity to learn (Mason et al., 2014). The effects of lead intoxication on memory have been amply demonstrated in animal models (Kuhlmann et al., 1997; Barkur et al., 2011). While neurones are sensitive to the toxicity of lead, they preferentially accumulate it in lysosomes, whereas astrocytes accumulate lead at a higher rate than neurones and concentrate it in the nucleus and throughout the cytoplasm (Holtzman et al., 1987). The results of the present study support the conclusion that the impairment of learning and memory in lead intoxication is due to the inactivation of astrocytic GS. Since the inactivation of GS by lead appears to be a secondary consequence of inhibition of the GSH system, it might be possible to reduce the effects of lead on cognition by providing supplements that boost the activity of the GSH system. Indeed, (Penugonda and Ercal, 2011) have shown that when lead-intoxicated rats are provided with N-acetylcysteine, a precursor of GSH, the depletion of GSH in the brain and other tissues is reversed. However, it remains to be shown whether such supplements can lessen the deficits in learning and memory caused by lead intoxication in rats and humans.

## Author Contributions

SRR designed and supervised the research project, reviewed the data analysis and was the lead author of the manuscript. AL undertook the experiments, collected and analyzed the data and drafted an early version of the manuscript. GMB supervised the research, assisted with data analysis and drafting of the manuscript. HC trained AL in the analytical techniques used, assisted with the preparation of figures and drafting the manuscript. RD provided technical advice and assisted with drafting the manuscript.

## Conflict of Interest Statement

The authors declare that the research was conducted in the absence of any commercial or financial relationships that could be construed as a potential conflict of interest.
